# Medicine

**Published:** 1906-12

**Authors:** Carey Coombs


### Progress of the Medical Sciences.

### MEDICINE.

**The use of sodium citrate in the feeding of infants** was suggested thirteen years ago by Wright,[1] who saw the practical importance of the fact that precipitation of the calcium salts in cow's milk rendered the casein clot lighter, and retarded its formation. Instead of following previous experimenters in the quite impracticable use of sodium fluoride he employed citrate of soda, and found that the addition of .5 per cent. of this salt completely inhibited the coagulation of milk by rennet, without affecting the taste. It is interesting to note in passing that the same writer[2] has attributed the frequency of venous thrombosis in enteric fever to a diet exclusively composed of milk, and therefore rich in calcium salts. Here, also, he suggests the addition of citrate of soda to the milk in order to decalcify it. Solt,[3] of Riga, has turned this hæmostatic action of milk to practical account by giving it in large doses, by the rectum if necessary, in cases of hæmoptysis, hæmatemesis, and other forms of internal hemorrhage, with very satisfactory results.

Wright's suggestion as to the use of citrate of soda in infant feeding seems to have escaped notice until two years ago, when Poynton[4] published the results of his experience in the out-patient department at Great Ormond Street. A more recent paper[5] shows that further trial has only served to confirm his good impression as to the value of this measure. It is useful in (*a*) the milk dyspepsia of infancy, (*b*) the weaning of healthy children from human to cow's milk, (*c*) rapid increase of the proportion of milk in mixtures with various diluents, and (*d*) it is said to avert the occurrence of scurvy. In the treatment of gastro-enteritis it has proved useless.

Immediately after Poynton's first paper came confirmatory data from France. Variot[6] spoke highly of the results he had obtained by adding citrate of soda to the milk of infants with irritable stomachs; Guyader[7] had succeeded in checking the vomiting of a child two months old by the addition of a drachm of 1 in 60 solution of the salt to each feed; and Ausset[8] also added his testimony to its efficacy in eleven cases.

During the current year three other writers have brought forward successful results obtained in the treatment of a large number of infants by this method. Shaw,[9] of New York, found

[1] *Lancet*, 1893, ii. 194. [2] *Med.-Chir. Tr.*, 1903, lxxxvi. 1.

[3] *Therap. Monatsh.*, Oct., 1906. [4] *Lancet*, 1904, ii. 433.

[5] *Med. Press and Circ.*, 1905, cxxxi. 369. [6] *Presse méd.*, 1904, p. 679.

[7] *Arch. de Med. d. Enf.*, 1904, p. 715. [8] *Pediat. prat.*, April, 1905.

[9] *Arch. Pediat.*, 1906, xxiii. 161.

that of twenty-two infants suffering from marasmus, dyspepsia, &c., seventeen gained weight satisfactorily while dieted on citrated milk, while five lost ground. The vomiting of chronic gastric catarrh was controlled, and curds disappeared from the stools. In a series of test-tube experiments which prefaced his clinical trials he found no other salt so satisfactory as a decalcifying agent; also that addition of calcium chloride to citrated milk was followed by the re-appearance of the objectionable bulkiness and density of curd removed by citration. An admirable paper by Wynn[1] constitutes, perhaps, the most complete contribution to the subject. There are, he says, a few infants whose stomachs are incapable of digesting cow's milk, the curd of which is very readily formed and very densely packed. To this excessive readiness of coagulation three factors contribute: first, casein constitutes six-sevenths of the total protein of cow's milk as against one-half of that in human milk; second, the casein of cow's milk contains an indigestible element, paranuclein, absent from human milk; and, third, cow's milk contains .17 per cent. of calcium salts as against .03 per cent. in human milk. These errors are mitigated though not removed by dilution; the casein clot still proves too dense for many infants, and causes pain and vomiting, with loose curdy motions. At this point the mother either substitutes an undesirable food or waters the milk down till it is quite insufficient for the needs of the child. Here it is that citrate of soda becomes useful; by precipitating calcium citrate it produces a slower coagulation and a more flocculent curd. The calcium thrown down is not, however, lost to the infant, but is presumably converted in the stomach into the readily soluble chloride. Wynn prescribes the salt according to Poynton's method, in aqueous solution protected from the growth of moulds by the addition of a few drops of spirits of chloroform. It is given in doses of from one to three grains per ounce of milk; the minimum dose is usually enough to begin with. The prescription should be so framed as to leave no calculation to the tender mercies of the mother or nurse; a simple method is to order a solution of such strength that one drachm has to be added to each feed. For example, if each feed consists of two ounces each of milk and water, the solution prescribed should contain two grains of citrate of soda per drachm. Wynn's cases, sixty-nine in number, fall into two categories: in the first, pain, wasting, vomiting and curdy stools are the indications for treatment, while the other class consists of children enfeebled by a diet of milk which has been excessively diluted to prevent its being "too strong for the baby's stomach." He has also used it in the weaning of healthy children, and in adults with gastric ulcer, pneumonia, and other diseases where plain cow's milk was not well borne.

Lastly, there is a paper by Cotton,[2] who has treated fifty

[1] *Birmingh. M. Rev.*, 1906, n.s. vii. 123. [2] *J. Am. M. Ass.*, 1906, xlvii. 1080.

infants with citrate of soda. Vomiting was checked, the stools became firm and free from curd (indeed, slight constipation is apt to give trouble), and a higher percentage of milk was tolerated. The method should not be abruptly discontinued, but gradually, by fractional reductions of the dose; the return of indigestion may call for resumption of the treatment. In only six of his cases did the method fail. From a small experience the writer of this abstract can bear witness to the value of the use of citrate of soda, particularly in the milk dyspepsia of feeble infants; there may be need for perseverance in spite of what seems to be a failure, for cases which are at first refractory will nevertheless yield to the treatment after a few days' persistence.

**The capacity of infants to digest starch** has recently occupied the attention of several American writers. Kerley and Mason[1] gave barley gruel (barley flour boiled for an hour and a half) to sixty children all under a year old, partly as a diluent and partly as a substitute for milk. In all 324 analyses of the stools were made. In thirty-three cases the fæces never contained starch, while in seven starch was always present; and the writers conclude that of the sixty infants forty-one showed a good capacity for digesting starch, while nineteen were poor in this respect. Some of the starch may, of course, have been destroyed by fermentation, but this cannot have taken place to any great extent, for there was no great abdominal distension; and, further, the presence of dextrin in the stools of more than half the cases proved that at any rate a partial starch digestion had occurred. The same writers, together with H. A. Craig,[2] publish the results of examination of the stools of twenty-six breast-fed infants for the presence of a starch enzyme, twenty-two of them being under a fortnight old. In all, 161 examinations of fæces were made, with the result that the stools of every infant proved to contain something which converted starch into maltose; on an average $\frac{1}{20}$ grain of starch was converted by 1 grain of fæces. That this was not due to bacterial action was proved by filtering the fæces after agitation with normal saline and using the filtrate, proved sterile by cultivation, for the starch experiments; it had a very definite diastatic power. Stools passed before breast-feeding began contained the ferment, so it is not derived from the maternal milk. Extract of macerated pancreas from infants a day old yields a starch diastase (Moro), so the inference is that the average infant secretes a starch-converting ferment from the earliest days. Indirect confirmation is given to this supposition by the work of Lopez,[3] who fed twenty-five infants between two and six months of age with foods containing varying percentages of starch, and also with controls consisting

[1] *Med. Rec.*, 1906, lxix. 989.
[2] *Arch. Pediat.*, 1906, xxiii. 489. [3] *Med. Rec.*, 1906, lxx. 495.

of modified cow's milk and a highly saccharated food containing converted starch. Briefly it may be said that a little of the starch appeared in the fæces of the infants fed on starchy food, that their digestion was not upset, and that they put on weight. The control foods produced vomiting, diarrhœa and loss of flesh, especially that containing much sugar. Lopez therefore concludes that "young infants can and do healthfully digest starch."

The discovery of the spirochæta pallida has given a fresh impetus to the study of **inherited syphilis**, and, among other points, to the investigation of the path of infection. Different workers studying different phases have found the organism in every stage of the way from mother to fœtus in utero. By Nattan-Larrier and Brindeau,[1] as well as by Wallich and Levaditi,[2] it has been seen in sections of the placentæ of infected women, lying in the connective tissue of the villi and also in the media of the arteries; none could be found in the thrombi of the uterine sinuses. More recently still, Ritter[3] has found the spirochæte traversing the wall of the umbilical artery of an infant born of a woman infected during pregnancy. Levaditi,[4] who made careful examinations of the organs of six infants dead of inherited syphilis, found spirochætes in great abundance in various parts of the body, but particularly in the liver, the organ to which the maternal blood is first carried. Further, he found that in those organs where the infection was most intense the morbid changes were most definite, namely in the liver, where there was an interstitial inflammation, in the lungs, the seat of "white pneumonia," in the adrenals, which had undergone "hypertrophy," and in the skin, where bullous and other eruptions were seen. Not only are the connective tissue and the blood-vessels attacked, but also the parenchyma of glandular organs such as the liver and kidneys. In the liver, indeed, spirochætes were in one case found engulfed within the hepatic cells; this observation derives peculiar interest from the fact that it was the only case examined in which the manifestations of syphilis had been delayed. The child, though born of luetic parents, was well till the age of two months, when a bullous eruption appeared and the child rapidly wasted, dying after a fortnight's illness. *Post-mortem*, spirochætes were found in the heart's blood, though a fortnight before they had been absent from blood taken from the finger; and, although they were plentifully sown throughout the liver, they were scattered through other organs much less thickly than in the other infants who died at or soon after birth. Levaditi therefore suggests that the delayed manifestations of syphilis are caused by infection localised to the tissues principally affected, while the acute

[1] *Progrès med.*, 1906, xxii. 86.

[2] *Ibid.* [3] *München. med. Wchnschr.*, 1906, liii. 2004.

[4] *Ann. de l'Inst. Pasteur*, 1906, xx. 41.

symptoms which lead to an early death are due to an acute dissemination of the spirochætæ throughout the body. Since the intensity of the morbid changes met with is in direct proportion to the number of spirochætæ present in the tissues, and since the organs are so thoroughly permeated by the infection, Levaditi concludes that the histological results produced by the organism are immediate and that no intermediary factor such as the diffusion of toxins need be called in to explain them. Two other points of practical importance were the observation of a phagocytic defence carried on by the macrophages of the pulmonary alveoli, and the presence of spirochætes in the kidneys and bronchi. From these latter observations it seems likely that the urine and sputa are potential carriers of infection.

Tobler,[1] stimulated by the discoveries of a definite lymphocytosis of the cerebro-spinal fluid in cases of tabes dorsalis, has performed lumbar puncture in seventeen cases of hereditary syphilis ranging from a few days to ten years of age. In fifteen of these a definite and unequivocal lymphocytosis was present, in two of them before any other clinical manifestations of the disease made their appearance; it is therefore possible that in doubtful cases a resort to lumbar puncture would prove a useful guide to early diagnosis and treatment by specifics.

CAREY COOMBS, M.D.

[1] *München. med. Wchnschr.*, 1906, liii. 1890.

